# Hepatocellular Carcinoma Treatment with Immune Checkpoint Inhibitors: RECA and CRAFITY Scores Reveal Distinct Clinical Courses and Highlight the Role of Systemic Inflammation in Prognosis

**DOI:** 10.3390/biomedicines14051043

**Published:** 2026-05-03

**Authors:** Xavier Adhoute, Constance Chailloux, Feng Xia, Zhao Huang, Qian Chen, Jing Yan, Qiao Zhang, Victoria Ramdour, Louis Carmarans, Guillaume Pénaranda, Paul Castellani, Albert Tran, Marc Bourlière, René Gerolami, Rodolphe Anty

**Affiliations:** 1 Department of Gastroenterology and Hepatology, Hôpital Saint-Joseph, 13000 Marseille, France; constancechailloux@gmail.com (C.C.); pcastellani@hopital-saint-joseph.fr (P.C.); mbourliere@hopital-saint-joseph.fr (M.B.); 2 Department of Hepatobiliary and Pancreatic Surgery, Tongji Hospital, Tongji Medical College of Huazhong University of Science and Technology, Wuhan 430000, China; 3 Department of Hepatobiliary Surgery, the First Affiliated Hospital of Shihezi University, Shihezi 832000, China; 4 Department of Ultrasound in Medicine, The Second Affiliated Hospital of Zhejiang University School of Medicine, Hangzhou 310000, China; 5 Department of Hepatic Surgery, Zhongshan People’s Hospital Affiliated with Guangdong Medical University, Zhongshan 528400, China; 6 Department of Hepatology, Hôpital Universitaire de l’Archet, 06000 Nice, Franceanty.r@chu-nice.fr (R.A.); 7 Department of Hepatology, Hôpital Universitaire de la Timone, 13000 Marseille, France; lcarmarans@gmail.com (L.C.);; 8 Department of Biostatistics, AlphaBio Laboratory, 13000 Marseille, France; guillaume.penaranda@biogroup.fr

**Keywords:** hepatocellular carcinoma, RECA, CRAFITY, systemic inflammation, immunotherapies, prognosis

## Abstract

**Background/Objectives**: Systemic treatment of advanced hepatocellular carcinoma (HCC) is based on combinations of immunotherapies (ITs) and lacks predictive markers of efficacy. Objectives: To define the prognostic value of the CRAFITY and RECA biological scores for overall survival (OS) before and during IT, and to evaluate the value of these two models for predicting the therapeutic response. **Patients and methods**: This was a multicenter retrospective analysis of 229 patients. OS was analyzed using Kaplan–Meier curves, log-rank tests, and Cox models, through which second-line therapy was modeled as a time-dependent covariate to avoid immortal time bias. The predictive capacity was assessed using univariate logistic regression. Validation was performed within two external Chinese cohorts. **Results**: Sixty-six percent of patients had Barcelona Clinic Liver Cancer (BCLC) stage C HCC (vascular invasion: 36.3%, metastases: 32.6%). After a mean follow-up of 14.9 (12.8) months, the median OS was 17.4 (6.9–38.0) months. The CRAFITY score distinguished only two different prognostic subgroups before treatment, but its prognostic value was confirmed with three different prognostic groups after 3 and 5 cycles and 6 months of treatment. The RECA score was strongly associated with OS before treatment and after 3 and 5 cycles and after 6 months of IT. Conversely, neither score had a discriminatory ability to predict early therapeutic response. The prognostic value of both models for OS was confirmed in the external cohorts. **Conclusions**: The RECA and CRAFITY scores have strong prognostic value for OS during IT. Beyond the models, the dynamic effects of systemic inflammation on IT reveal distinct clinical outcomes. Neither score has the ability to predict early therapeutic response, further supporting their use during treatment.

## 1. Introduction

Management of hepatocellular carcinoma (HCC), the leading cause of primary liver cancer, is challenging because of underlying cirrhosis, delayed diagnosis despite well-identified risk factors [1], and failure to consider histology or molecular classifications [2]. Ultimately, the overall prognosis remains poor. However, significant changes and progress have been made in recent years, such as in the introduction of the ALBI grade [3], in the stratification of intermediate stages [4] and in treatments such as intra-arterial procedures [5] or systemic therapies with the advent of immune checkpoint inhibitors (ICIs). Therefore, from 2020 onward, several antibody combinations targeting the immune checkpoints human cytotoxic T-lymphocyte antigen-4 (CTLA-4), programmed death receptor-1 (PD-1) or its ligand PD-L1 or a combination of ICIs and monoclonal antibodies targeting vascular endothelial growth Factor A (VEGF-A) became the standard of care in advanced stages, surpassing treatment with tyrosine kinase inhibitors (TKIs) [6,7,8,9,10]. However, radiological responses are observed in only one-third of patients, with frequent acquired resistance. Following the same rationale of combining drugs given the limited response rate for monotherapy, triple associations combining two ICIs with an anti-VEGF-A antibody [11,12] were evaluated but did not show any additional benefit. Approximately 30% of HCCs belong to an “immune” class, defined by significant infiltration of immune cells strongly involved in the mechanism of action of ICIs [13]. To date, there are no approved predictive biomarkers for the response to immunotherapy in patients with HCC. Inflammation and tumor necrosis are closely intertwined and promote tumor growth [14]. The presence of systemic inflammation is a poor prognostic factor in patients with cirrhosis with or without cancer, through immunoparesis and metabolic dysfunction [15]. Serum inflammatory markers such as the neutrophil-to-lymphocyte ratio (NLR) and C-reactive protein (CRP) have been shown to have prognostic value for survival in patients with HCC [16,17]. Biological inflammatory scores including the CRAFITY [18] (based on serum levels of CRP and alpha-fetoprotein (AFP)) and RECA models [19] (based on serum levels of CRP, aspartate aminotransferase, (AST), and the NLR) have shown prognostic value for survival primarily prior to treatment and during the course of ICI treatment, respectively. These simple models could therefore reveal distinct clinical outcomes and provide additional support and guidance to clinicians in assessing prognosis and cancer status. However, the most appropriate time for calculating these scores remains to be determined since immunotherapy combinations’ efficacy does not correlate with tumor burden, and the restoration of antitumor immunity affects both AFP levels [20] and systemic inflammation [21].

The aims of this multicenter study, which was conducted in real-life settings, were as follows:

1. To describe the characteristics of patients with (locally) advanced HCC who had received at least one line of ICIs and their treatment regimens and to evaluate the efficacy of treatments in terms of OS.
2. To analyze the prognostic value of the RECA and CRAFITY models for OS before and during treatment with ICIs and to evaluate the predictive value of these two models for assessing radiological response.

## 2. Materials and Methods

This multicenter retrospective study was performed at three institutions in southern France (Hôpital Saint-Joseph Marseille (HSJ), Centre Hospitalo-Universitaire (CHU) de la Timone (T) in Marseille, and CHU l’Archet (A) in Nice) from September 2020 to June 2025. The medical records of all consecutive patients who were diagnosed with (locally) advanced HCC according to the Barcelona Clinic Liver Cancer (BCLC) staging system and who were treated with ICIs alone or in combination with or without angiogenesis-targeting agents were considered for the study. The diagnosis of HCC was based on radiology in accordance with international guidelines [22] or on histology in the absence of formal radiological criteria. The therapeutic decision followed a multidisciplinary team discussion in accordance with local practices at each center. The selected patients included those with BCLC stage B HCC without curative options, namely, evolutive multinodular HCC refractory to transarterial chemoembolization (TACE), or BCLC stage C HCC displaying vascular invasion and/or metastatic disease; Eastern Cooperative Oncology Group (ECOG) performance status (PS) 0/1 or even 2 according to each institution, and liver function classified as Child–Pugh (CP) grade A or even B according to each institution. The data were collected prospectively through an internal database within each center and analyzed retrospectively. The data included baseline data before starting ICI therapy and follow-up demographic, clinical, and biological characteristics, including full blood count, biochemical blood tests (particularly alpha-fetoprotein (AFP) and CRP levels) and radiological features. Upper gastrointestinal endoscopy was performed prior to treatment in patients with cirrhosis or prior to the use of angiogenesis-targeting therapeutic agents. Only patients for whom critical data were available were included. A total of 229 consecutive patients with analyzable data were included (HSJ *n* = 142; A *n* = 59; T *n* = 28). To best evaluate our results on the prognostic value of the RECA and CRAFITY scores, two Chinese multicenter external validation cohorts were used in our series. These cohorts included 410 patients (CRAFITY score; RECA, *n* = 332) with (locally) advanced HCC treated with ICI combinations during the same period.

### 2.1. Protocols

#### 2.1.1. Standard Treatment Regimens

- Patients received 1200 mg atezolizumab (Atz) (anti-PDL1 antibody) every 3 weeks plus 15 mg/kg bevacizumab (Bev) (anti-VEGF-A antibody) every 3 weeks intravenously according to the IMbrave-150 study [6].
- The combination of durvalumab (Durv) (anti-PDL1 antibody) plus tremelimumab (Trem) (anti-CTLA-4 antibody) was administered according to the STRIDE regimen [7]: tremelimumab was administered only during the first course of treatment (300 mg), and durvalumab was administered every 4 weeks at a dose of 1500 mg.
- The combination of nivolumab (anti-PD1 antibody) at 1 mg/kg plus ipilimumab (anti-CTLA-4 antibody) at 3 mg/kg was administered every 3 weeks for 4 cycles (induction phase), followed by nivolumab alone (480 mg) every 4 weeks (maintenance phase) [8].

#### 2.1.2. Treatment Regimens in Clinical Trials

- Patients received either 600 mg of anti-TIGIT monoclonal antibody plus 1200 mg of Atz plus 15 mg/kg of Bev or 1200 mg of Atz plus 15 mg/kg of Bev, which was administered via intravenous infusion every 3 weeks on Day 1 of each 21-day cycle.
- Patients received either 1200 mg of Atz plus 15 mg/kg Bev plus 1 mg/kg Ipilimumab every 3 weeks (4 doses) followed by the Atz plus Bev combination every 3 weeks or 1200 mg of Atz plus 15 mg/kg Bev administered via intravenous infusion every 3 weeks on Day 1 of each 21-day cycle.
- Patients received monotherapy with an anti-PD1 antibody at a dose of 200 mg every 3 weeks.

Patients were informed of potential adverse events (AEs) before starting treatment. Treatment was continued until radiological progression and/or loss of clinical benefit or until the onset of serious adverse events. The occurrence of AEs was assessed at each reassessment and classified according to the National Cancer Institute (NCI CTCAE) v5.0 terminology.

Procedure and assessments: Follow-up included a clinical evaluation prior to each cycle. Radiological assessment included initial cross-sectional imaging (computed tomography and/or magnetic resonance imaging) approximately ten to twelve weeks after the start of treatment and then every three months using the Response Evaluation Criteria in Solid Tumors (RECIST) version 1.1 for the grading of tumor responses. Patients with controlled disease included those with radiological response (RR) and stable disease (SD) as the best response. Liver function (albumin and bilirubin), liver tests (alanine aminotransferase, ALT; AST; alkaline phosphatase, ALP; γ-glutamyl transpeptidase, GGT), AFP, and inflammation-related factors (NLR and CRP) were also evaluated during each treatment cycle.

The CRAFITY score is a point-based score based on serum AFP levels (if ≥ 100 ng/mL, one point is assigned) and serum CRP levels (if ≥ 1 mg/dL, one point is assigned); the results are therefore 0, 1, or 2 (low, intermediate, or high CRAFITY score, respectively).

The RECA score is calculated using the following formula: −0.1849 + 0.1943 × (1 if NLR > 3 and 0 if ≤3) + 0.3053 × (1 if CRP > 10 [mg/L] and 0 if ≤10) + 0.4962 × (1 if AST > 45 [IU] and 0 if ≤45): https://jscalc.io/calc/3nzmguiJK5QIn8eQ#%7B%221%22:null,%222%22:null,%223%22:null%7D (accessed on 15 August 2022).

**Figure 1:**
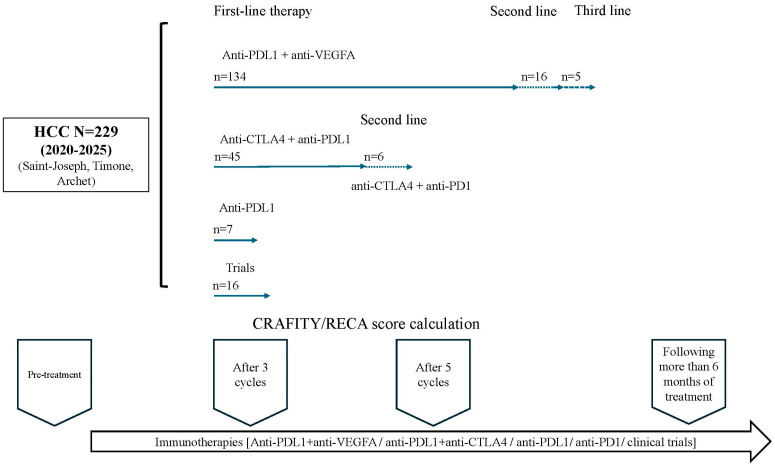
Treatment regimens in the French cohort and RECA/CRAFITY score schedule.

The CRAFITY and RECA scores were calculated prior to treatment initiation (based on biological data collected 24 h before), after the third and fifth cycles of immunotherapy (based on biological data collected 24 h prior to the fourth and sixth cycles, respectively) and more than six months after treatment initiation (between the seventh and ninth months) (Figure 1). The scores were calculated after the fifth cycle of immunotherapy regarding the Chinese validation cohorts (based on biological data collected 24 h prior to the sixth cycle).

### 2.2. Statistics

Quantitative data are summarized using means and standard deviations (SDs), whereas qualitative variables are described using sample sizes and percentages. Comparisons between groups for quantitative variables were performed using Student’s test when the data followed a normal distribution or Wilcoxon’s nonparametric test when they did not (normality testing was performed using Shapiro–Wilk test). Qualitative variables were compared using the chi-square test or Fisher’s exact test, depending on the conditions of applicability. For ordinal categorical variables, the Mantel–Haenszel chi-square test was used.

In this cohort, some patients received a second-line treatment during follow-up, while others remained on first-line treatment until the end of the observation period. A standard analysis treating the second line as a fixed variable would have introduced an immortal time bias, as patients accessing a second line would necessarily have to survive until its initiation. To avoid this distortion and accurately reflect the actual evolution of therapeutic exposure, a model considering the temporal nature of this variable was applied [23,24,25,26]. Second-line status was therefore modeled as a time-dependent covariate, structuring the data in the form of start–stop intervals. Each patient contributed to the risk, with TREAT2 = 0 until the eventual initiation of the second-line therapy and then with TREAT2 = 1 after that date. This approach correctly attributes exposure to the moment it occurs and thus eliminates the immortal time bias that would arise if the second line were treated as a fixed characteristic. The Cox model thus enables the estimation of the specific effect of the prognostic score studied, adjusted for the actual evolution of therapeutic status during follow-up.

Overall survival (OS) was defined as the time between HCC diagnosis and death from any cause or the date of last follow-up for censored patients. Survival comparisons were performed using the log-rank test, and OS is reported as the median and interquartile range (Q1, Q3). A Cox proportional hazards regression model was used to estimate hazard ratios (HRs) and their 95% confidence intervals (CIs).

The associations between RECA scores (quantitative variable) and CRAFITY scores (ordinal variable, reference = 0) and the clinical criteria studied—objective response to treatment (complete response + partial response)—were evaluated using univariate logistic regressions. For each assessment point (after the third cycle, after the fifth cycle and beyond the sixth month of treatment), separate models were developed to examine the predictive ability of scores measured before treatment or re-evaluated during follow-up (after the third cycle and the fifth cycle). The results are presented as odds ratios (ORs) with 95% confidence intervals and associated *p* values. For models built from the RECA score, discriminant performance was also estimated using the area under the ROC curve (AUC) and its confidence interval. A two-tailed *p* value < 0.05 was considered to indicate statistical significance. All the statistical analyses were performed using SAS software, version 9.4 (SAS Institute Inc., Cary, NC, USA).

## 3. Results

### 3.1. Patient Characteristics (French Cohort)

The mean age was 69.4 years and the population included mostly men with cirrhosis (Table 1), predominantly those classified as Child–Pugh (CP) grade A (83.6%). More than one-third of the patients had clinical significant portal hypertension and 86.5% of them received preventive medical treatment with beta-blockers and/or instrumental therapy. Sixty-six percent of patients had BCLC stage C HCC (vascular invasion: 36.3%, metastases: 32.6%), while the other patients had intermediate-stage HCC. More than half of the patients had received one or more prior locoregional treatments and 10% had received systemic treatment with TKIs.

In this series, 88% (*n* = 202) of patients (Figure 1) received systemic treatment with ICIs as first-line therapy, mainly as a combination (91.5%, *n* = 185). The Atz + Bev regimen was the most frequently administered combination as first-line therapy (66.3%, *n* = 134), followed by the Durva + Trem regimen (22.2%, *n* = 45). Patients underwent a median of 6 cycles of ICIs (Q1–Q3: 4–13).

Patients from the Chinese cohorts mostly had advanced HCC (60.7–72.6%), which was predominantly linked to the hepatitis B virus (72.3–76.8%) (Supplementary Table S1). Their treatment regimens included combinations of ICIs with or without angiogenesis-targeting agents (Supplementary Table S2).

### 3.2. Efficacy

#### 3.2.1. French Cohort

After a mean follow-up of 14.9 months (±12.8), 56.7% of the patients died, and the median OS for the entire cohort was 17.4 (6.9–38.0) months. OS was significantly longer for patients classified as CP grade A than for those classified as CP grade B [20.9 (8.3–40.1) vs. 9.1 (4.1–16.7) months, respectively, HR = 2.36 (95% CI: 1.48–3.76), *p* = 0.0003]. OS was also greater for patients classified as ALBI grade 1 than for those classified as ALBI grades 2 and 3 [31.2 (13.2–inf) vs. 12.8 (5.6–26.7) months, respectively, HR = 2.57 (95% CI: 1.71–3.86), *p* < 0.0001; 31.2 (13.2–inf) vs. 9.6 (5.2–14.2) months, HR = 4.42 (95% CI: 1.70–11.51), *p* = 0.0023]. There was no significant difference in OS according to first-line combination therapy [Atz + Bev: 16.7 (6.8–40.1) vs. Durva + Trem: 22.7 (22.7–inf) months, HR = 0.49 (95% CI: 0.22–1.06), *p* = 0.0695] or according to the presence or absence of clinical significant portal hypertension [12.0 (5.4–inf) vs. 20.9 (8.7–51.8) months, respectively, HR = 1.22 (95% CI: 0.80–1.88), *p* = 0.3580].

A radiological response was observed in 38 of 148 evaluable patients (65% of the population) at 10–12 weeks, resulting in an RR of 26% (including 10.5% with CR) according to the RECIST 1.1 criteria and a DCR of 68%, with disease progression observed in 32% of patients (*n* = 47). A response was observed in 41 of 148 evaluable patients (65% of the study population) after 24 weeks, resulting in an RR of 28% (including 5% with CR) and a DCR of 72%, with disease progression observed in 28% of patients (*n* = 41).

**Figure 2:**
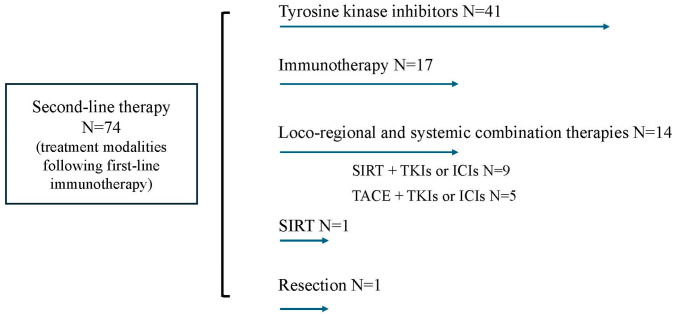
Second-line treatment modalities in the French cohort.

Thirty-two percent of patients (*n* = 74) received second-line therapy (Figure 2), mainly systemic treatment with TKIs (*n* = 41) or with ICIs (*n* = 17), whereas the others received a combination of local-regional and systemic therapy (TACE + TKIs or ICIs; *n* = 5; SIRT + TKIs or ICIs; *n* = 9). One patient was treated with resection surgery, and another was treated with SIRT as second-line therapy.

Adverse effects reported in the French cohort are listed in the Supplementary Table S5.

#### 3.2.2. Chinese Cohorts

The median overall survival times observed in the validation cohorts were 20.8 months (RECA analysis cohort, *n* = 332) after a median follow-up duration of 23.9 months, and 18.4 months (CRAFITY analysis cohort, *n* = 410) after a median follow-up duration of 25.6 months. In addition, 43.1% of patients in the RECA cohort and 43.7% of patients in the CRAFITY cohort received subsequent systemic therapy.

### 3.3. Prognostic Performance of the CRAFITY and RECA Scores for Overall Survival

#### 3.3.1. CRAFITY

**Figure 3:**
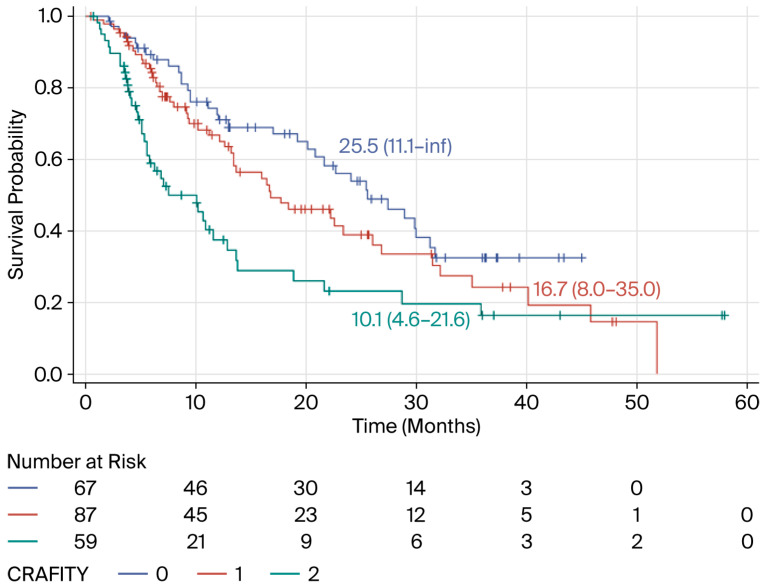
Kaplan–Meier curves describing the overall survival of HCC patients treated with combinations of ICIs with or without anti-angiogenic agents according to the CRAFITY score before treatment initiation (French cohort).

The calculation of the score before treatment initiation revealed that, according to the Cox model, compared with patients with a CRAFITY score of 0, patients with a CRAFITY score of 1 had a nonsignificant increase in the risk of death [OS: 25.5 (11.1–inf) vs. 16.7 (8.0–35.0) months, respectively, HR = 1.39 (95% CI: 0.89–2.16); *p* = 0.1457], and those with a CRAFITY score = 2 had a significantly increased risk [25.5 (11.1–inf) vs. 10.1 (4.6–21.6) months, respectively, HR = 2.29 (95% CI: 1.43–3.66); *p* = 0.0006] (Figure 3). Furthermore, in this adjusted Cox model, there was a negative effect associated with the initiation of second-line therapy (TREAT2) [HR = 1.50 (95% CI: 1.03–2.20); *p* = 0.0371], indicating an increased risk of death unrelated to the prognosis estimated by the CRAFITY score.

**Figure 4:**
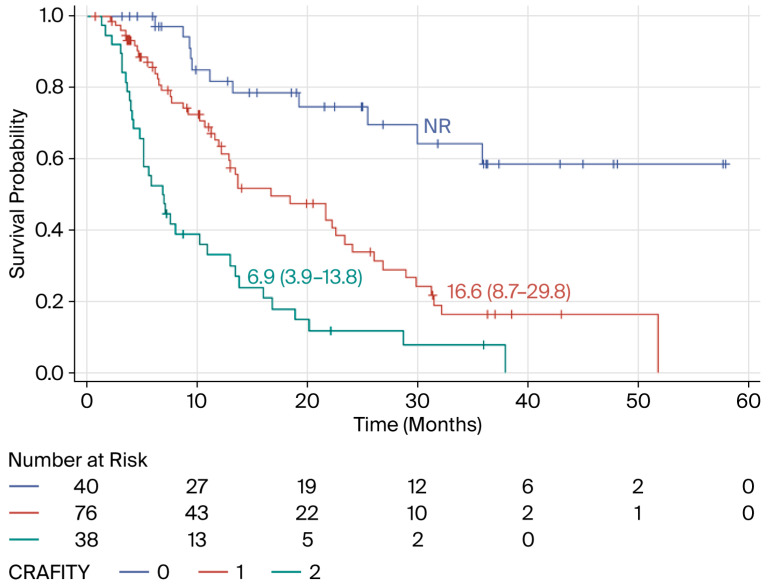
Kaplan–Meier curves describing the overall survival of HCC patients treated with combinations of ICIs with or without anti-angiogenic agents according to the CRAFITY score after three cycles of treatment (French cohort).

Following the third cycle of ICI treatment, the CRAFITY score indicated three different prognostic groups (Figure 4). Compared with patients with a CRAFITY score of 0, patients with a CRAFITY score of 1 had a 3.32-fold increased risk of death (95% CI: 1.71–6.45) (*p* = 0.0004), and those with a CRAFITY score of 2 had a 7.67-fold increased risk (95% CI: 3.84–15.33) (*p* < 0.0001). According to this adjusted Cox model, the initiation of second-line therapy (TREAT2) was not independently associated with survival [HR = 1.14 (95% CI: 0.73–1.77); *p* = 0.5638].

**Figure 5:**
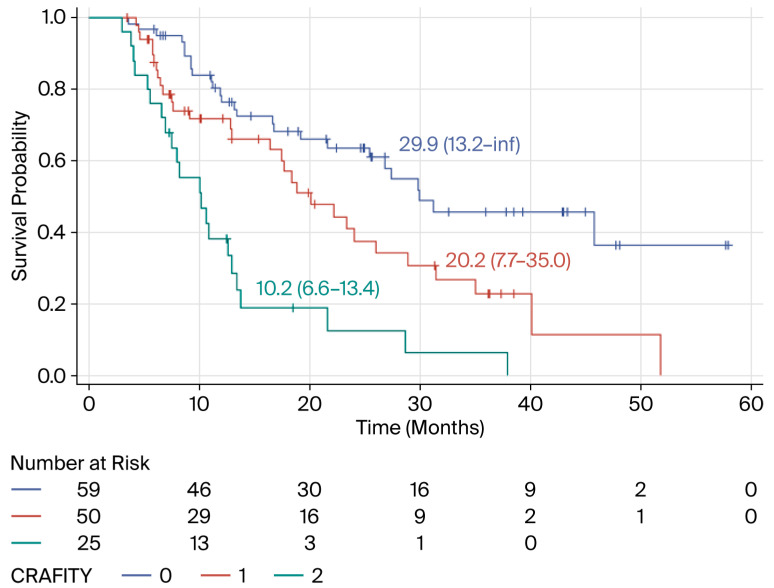
Kaplan–Meier curves describing the overall survival of HCC patients treated with combinations of ICIs with or without anti-angiogenic agents according to the CRAFITY score calculated after five cycles of treatment (French cohort).

The calculation of the score after the fifth cycle revealed that, according to the Cox model, compared with the CRAFITY = 0 group, patients with a CRAFITY score of 1 had a 1.37-fold increased risk of death (95% CI: 1.16–3.35) (*p* = 0.0120), whereas patients with a CRAFITY score of 2 had a 4.72-fold increased risk (95% CI: 2.62–8.50) (*p* < 0.0001) (Figure 5). Switching to second-line treatment was not independently associated with OS [HR = 1.31 (95% CI: 0.83–2.06); *p* = 0.2568]. Beyond the sixth month, the risk of death still progressively and markedly increased according to the score level. Compared with the CRAFITY = 0 group, patients with a CRAFITY score of 1 had a 3.75-fold increased risk of death (95% CI: 1.86–7.56) (*p* = 0.0002), whereas patients with a CRAFITY score of 2 had a 9.23-fold increased risk (95% CI: 4.29–19.9) (*p* < 0.0001). In this adjusted Cox model, switching to second-line treatment was not independently associated with survival [HR = 0.95 (95% CI: 0.58–1.53), *p* = 0.8176]. Flowchart of HCC patients from the French CRAFITY cohort can be found in Supplementary Figure S1. 

**Figure 6:**
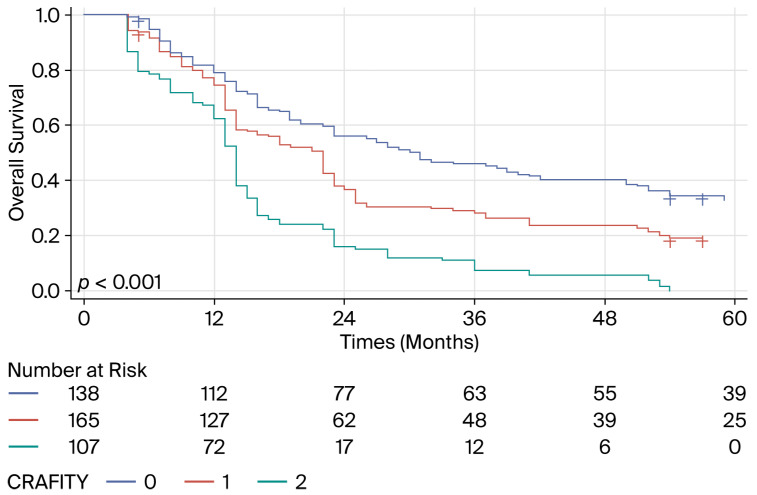
Kaplan–Meier curves describing the overall survival of HCC patients treated with combinations of ICIs with or without anti-angiogenic agents according to the CRAFITY score calculated after five cycles of treatment (Chinese cohort).

In the external validation cohort, the CRAFITY score, which was calculated following five courses of immunotherapy, revealed three distinct prognosis groups with significant differences in OS (Figure 6).

#### 3.3.2. RECA

In all Cox models, the RECA score showed good prognostic performance for OS, both at inclusion and throughout treatment.

Prior to treatment initiation, the RECA score, regarded as a continuous variable, was strongly associated with OS; a 1-point increase in the RECA score (corresponding approximately to the total range observed in the cohort) was associated with a threefold increase in the risk of death [HR = 3.01 (95% CI: 1.85–4.87), *p* < 0.0001]. An increase of 0.1 to 0.2 points in the RECA score was associated with an estimated increase in the risk of death ranging from 12% to 25%, supporting the linear progression of risk with increasing score. Switching to second-line treatment increased the risk of death [HR = 1.57 (95% CI: 1.07–2.31), *p* = 0.0217], indicating an increased risk unrelated to the prognostic profile measured by the RECA model.

After the third cycle, the RECA score was strongly associated with OS. A one-unit increase in the RECA score was associated with a 4.62-fold increase in the risk of death (95% CI: 2.83–7.55; *p* < 0.0001). A variation of +0.1 points corresponded to an approximately 16% increase in the risk of death, reflecting a continuous and marked prognostic gradient. In the same model, switching to a second-line treatment was associated with a nonsignificant increase in the risk of death [HR = 1.47 (95% CI: 0.99–2.18); *p* = 0.0557]. The same result was observed after the fifth cycle and beyond the sixth month of treatment, with the RECA score strongly associated with OS; a one-unit increase in the RECA score was associated with a 5.74-fold (95% CI: 3.19–10.35) (*p* < 0.0001) and a 5.18-fold (95% CI: 2.72–9.87) (*p* < 0.0001) increase in the risk of death, respectively. 

**Figure 7:**
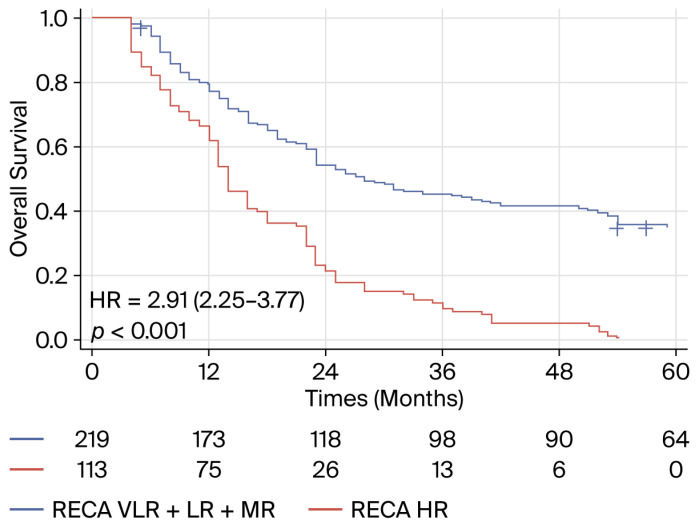
Kaplan–Meier curves describing overall survival of HCC patients treated with combinations of ICIs with or without anti-angiogenic agents according to the RECA score calculated after five cycles of treatment [<0.15 (very low risk of progression) + 0.15–0.40 (low risk) + 0.40–0.60 (moderate risk) versus > 0.60 (high risk)] (Chinese cohort).

In the external validation cohort, the RECA score was categorized into two groups [<0.15 (very low risk of progression) + (0.15–0.40) low risk + (0.40–0.60) moderate risk versus (>0.60) high risk] [19]. After five cycles of treatment, two groups with different prognoses were identified (Figure 7).

A further analysis in both validation cohorts using multivariable Cox models including age, sex, Child–Pugh grade, BCLC stage, macrovascular invasion, extrahepatic metastases, treatment category and subsequent therapy, revealed that the RECA and the CRAFITY scores remained independently associated with OS (RECA high-risk group versus very low risk/low risk/moderate risk groups: adjusted HR = 2.31 (95% CI: 1.74–3.06) (*p* < 0.001); CRAFITY score 1 versus. 0: adjusted HR = 1.49 (95% CI: 1.12–1.98) (*p* = 0.006); score 2 versus 0: adjusted HR = 2.47 (95% CI: 1.76–3.47) (*p* < 0.001) (Supplementary Table S3; Supplementary Figure S2).

#### 3.3.3. Predictive Value of the CRAFITY and RECA Scores for Treatment Response

The CRAFITY score calculated before treatment had no predictive value for an early response (after the third cycle). Compared with patients with a CRAFITY score of 0, patients with a CRAFITY score of 1 were nearly three times more likely to be responders after the third cycle [OR = 2.93 (1.06–8.13), *p* = 0.0391] (Table 2a); patients with a CRAFITY score of 2 were not significantly more likely to respond [OR = 2.74 (0.91–8.22), *p* = 0.0720]. Similarly, there was no association for subsequent cycles. Conversely, the CRAFITY score calculated after the third cycle had good predictive power for assessing the response after the fifth cycle. Patients with a CRAFITY score > 0 were significantly less likely to respond after the fifth cycle (Table 2b). The CRAFITY score calculated after the fifth cycle had no significant predictive value for response beyond the sixth month, but the OR value for a CRAFITY score of 2 suggested a decrease in the probability of response [OR = 0.13 (0.02–1.14), *p* = 0.0656] (Table 2c).

Thus, the CRAFITY score (measured after the third cycle) was the most informative, clearly surpassing the baseline score in predicting responses to treatment in the near term.

The RECA score calculated before treatment showed no discriminatory capacity for predicting an early response to treatment (after the third cycle). Patients who responded after three cycles had higher RECA scores prior to treatment than those who did not respond (Table 3a). Similarly, the score had no discriminatory ability to predict a response for subsequent cycles (after the fifth cycle and beyond six months of treatment). After the third cycle, the score had no discriminatory power; however, compared with those without a response, patients with a response after the fifth cycle had a lower RECA score after the third cycle, but the difference was not significant (Table 3b). The results for predicting response beyond six months of treatment, based on an assessment after the fifth cycle, are comparable (Table 3c).

Both models showed limited ability to predict early therapeutic response within the external validation cohorts (Supplementary Table S4).

## 4. Discussion

The OS results observed in our multicenter French cohort, which was mainly composed of advanced HCC patients with nonviral cirrhosis, confirm the benefits of ICIs combinations, with or without angiogenesis-targeting agents, over treatment with TKIs [27]. The OS, estimated at 17.4 months, was comparable to those reported in other real-world clinical series [28], the Chinese validation cohorts associated with this study, and phase III trials [7,29]. There were fewer patients with metastases in our cohort than in phase III trials (53–73%) [6,7,9], but vascular invasion was observed in more than one-third of patients, similar to real-world studies [28,30,31]. These results reflect the natural history of HCC, which has long been an intrahepatic disease [32]. In our cohort, more than 50% of patients had received prior locoregional treatment. Our cohort included 33.9% intermediate HCC cases, as in other series [33,34], particularly because of the improved stratification of BCLC B stages [4] and systemic treatment for diffuse or infiltrating HCC. Portal hypertension, which is a recognized prognostic factor [35], did not significantly affect OS in our study. These results should be interpreted with caution, as this is a retrospective series with a limited sample size and no matching. However, they are not completely surprising given the management of clinical significant portal hypertension, with 86.5% of patients receiving preventive therapy with beta-blockers and/or endoscopic treatment or even the presence of portosystemic shunts. Our cohort included 16.6% of patients with Child–Pugh grade B cirrhosis [28,30], who had a median OS of 9.1 months, similar to that reported in other real-world studies [33,34]. ICIs have a better safety profile than TKIs do in this particular population, and OS also appears to be better [36]. These patients constitute a heterogeneous population in clinical practice, and the ALBI score [3] allows for better stratification. Studies [37] are ongoing to further define which patients within this population should be treated. Combination therapies have become the standard treatment for advanced HCC [6,7,9,10,29,38]. In our cohort with no matching and a retrospective analysis, we found no significant difference in efficacy between a combination of ICIs and a combination of ICIs plus angiogenesis-targeting agents. Notably, the combination of Atz plus Bev has been available in our French centers since August 2020, whereas the combination of Durva plus Trem became available in 2023. This means that there were differences in the duration of follow-up. To date, there are no decisive criteria to guide clinicians’ choice for the treatment other than potential contraindications related to the use of antiangiogenic drugs.

Our results, from both the French and Chinese cohorts, support the prognostic value of the RECA and CRAFITY models for OS following systemic ICI therapy, with score kinetics significantly correlated with survival. By identifying patients with different prognoses under treatment, the RECA and CRAFITY scores enable clinicians to better assess patients’ overall cancer status and can therefore provide additional support for therapeutic adjustments or an earlier radiological evaluation. The prognostic capacity of both models for OS, measured at different intervals during treatment, remained consistent over time, supporting the value of regular calculation. Furthermore, our decision to include second-line therapy exposure in the analysis to avoid immortal time bias strengthens our conclusions regarding the prognostic performance of the models. The unfavorable nature of second-line therapy at baseline estimation is probably related to the limited efficacy of angiogenesis-targeted therapies, which were the main treatment administered, and/or to a more unfavorable clinical situation in patients who failed first-line systemic therapy with ICIs.

Our study further highlights the relevance of evaluating serum inflammatory markers (CRP and NLR) in advanced HCC [16,31,39] at different time points, as they reveal distinct clinical trends during treatment. The differences observed between the RECA and CRAFITY scores of patients during response and the baseline scores suggest that the decrease, or even normalization, in systemic inflammatory parameters reflects treatment efficacy, as suggested by preclinical studies [40,41]. These results should be interpreted with caution, as this is a retrospective series, but they are found in Asian patients with HCC of different etiologies and are not surprising. Tumor necrosis leads to the release of proinflammatory signals, protumor immune cell recruitment, and the promotion of angiogenesis driven by hypoxia. Myofibroblast enrollment and proteolytic enzyme activation enable tissue invasion, and cytokines such as interleukin (IL)-11 and IL-6 are secreted to support tumor growth through activation of the IL-6/NF-kB/STAT3 signaling cascade [42]. Activation of the STAT3 signaling protein plays a key role in inducing a procarcinogenic, immunosuppressive inflammatory microenvironment, particularly by preventing dendritic cell maturation and in inducing tumor angiogenesis and invasion via epithelial-mesenchymal transition. High IL-6 levels are associated with increased serum CRP levels [43]. Lymphopenia and neutrophil counts contribute to the oncogenic process, and both affect progression-free survival and OS in patients with many advanced solid tumors [44]. The prognostic value of the NLR is now strongly suggested in the context of ICI therapy, particularly during treatment [45].

The lack of predictive capacity of both models for early response is not surprising. As shown in our study, systemic inflammation and/or increased AFP levels [46] reflect an unfavorable oncological status for patients with (locally) advanced HCC, but they do not preclude a response to ICI therapy. We still do not have predictive biomarkers of efficacy for routine use. Immunohistochemical PD-L1 expression status in tumors and immune cells has been shown to be limited in HCC, with no significant correlation between its expression and survival [47]. The tumor microenvironment and some CD8 T-cell populations that infiltrate tumors are strongly involved in the action of ICIs and clearly constitute a promising approach. Tumor cells use various mechanisms to disrupt the immune response, either by evading recognition and/or by inducing a highly immunosuppressive tumor microenvironment, notably through the recruitment of suppressive myeloid cells and regulatory T cells, the expression of inhibitory coreceptors on T cells, and the reduction in functional dendritic cells [48]. Angiogenesis also contributes to immunosuppression via endothelial cells through the expression of the coinhibitory molecule PD-L1 or through a direct effect of proangiogenic factors such as VEGF-A [49]. Molecular biomarkers of the response to ICI therapy have emerged. Gene expression profiling of tumors involves the simultaneous measurement of the expression of several hundred genes involved in the immune response [50]. Recently, “enrichment” in CD8+ effector cells and CXCL10+ proinflammatory macrophages enabled the identification of a subgroup of “inflammatory tumors” in advanced HCC in response to the combination of Atz plus Bev. An activated beta-catenin signature was associated with a nonresponse to ICI therapy. Furthermore, “noninflammatory” tumors that responded to this combination had low expression of neuropilin-1, a VEGF-A coreceptor, suggesting a favorable response to bevacizumab [51].

Our study has several limitations including its retrospective nature, the lack of matching for comparative studies, the lack of centralized review of imaging for response assessment, and the lack of data on progression-free survival. The presence of an external foreign validation cohort helps to overcome some of these biases. The lack of predictive capacity for early therapeutic response is clearly a limitation to this type of score’s usefulness, particularly for therapeutic decision-making. The RECA model is a continuous-variable score that combines several inflammatory markers. Accordingly and for simplicity’s sake, we did not evaluate other inflammatory-related prognostic biomarkers. Furthermore, our study suggests rather than demonstrates a correlation between the dynamics of systemic inflammation and therapeutic response.

## 5. Conclusions

The results observed across all cohorts in this multicenter study confirm that OS benefits from the combination of ICIs, with or without angiogenesis-targeting agents as a treatment for advanced HCC. Our study revealed that the CRAFITY and RECA biological inflammation scores are reliable prognostic tools for OS during treatment with ICIs, supporting their regular use to accurately assess cancer status. Furthermore, whether systemic inflammation is controlled reveals a distinct clinical course for patients with HCC receiving ICI combinations. The ability of these two models to predict an early therapeutic response is absent in our series.

## Figures and Tables

**Table 1:** Patient characteristics prior to treatment by ICIs.

Baseline Characteristics		Available Data
Age—mean (Sd)	69.4 (9.2)	*n* = 229
BMI—mean (Sd)	27.1 (5.2)	*n* = 151
Gender—*n* (%) Male/Female		*n* = 229
	195 (85.2)/34 (14.8)	
Cirrhosis—*n* (%)	138 (81.6)	*n* = 229
Etiology of HCC—*n* (%)		*n* = 222
Alcohol/Virus/MASH/Other	59 (26.6)/48 (21.6)/45 (20.3)/27 (12.2)	
Alcohol + MASH/Alcohol + virus	18 (8.1)/25 (11.2)	
Child-Pugh Grade—*n* (%)		*n* = 229
A/B	191 (83.4)/38 (16.6)	
Esophageal varices—*n* (%)	74 (32.6)	*n* = 227
Significant portal hypertension	60 (35.5)	*n* = 169
Portal hypertension treatment	*n* = 64	
Beta blocker ± instrumental treatment	*n* = 48	
Sclerotherapy and/or biological glue	*n* = 12	
portosystemic shunt	*n* = 4	
ECOG performance status—*n* (%)		*n* = 227
0/1/2	103 (44.9)/108 (47.1)/18 (8.0)	
Pathology: Edmondson grade—*n* (%)		*n* = 165
1–2/3–4	112 (67.9)/53 (32.1)	
Maximal tumor diameter—mean (cm)	5.97 (3.66)	*n* = 204
Extrahepatic disease—*n* (%)	74 (32.6)	*n* = 229
BCLC—*n* (%)		*n* = 229
B	78 (33.9)	
C	151 (66.1)	
Macrovascular invasion—*n* (%)	82 (36.3)	*n* = 226
VP1/VP2	32 (14.2)	
VP3	22 (9.7)	
VP4	17 (7.5)	
Other	11 (4.9)	
No	144 (63.7)	
Previous locoregional treatment—*n* (%)		*n* = 222
	125 (56.3)	
Systemic treatment with TKIs	22 (10)	
Biological results		
Hemoglobin g/dL—mean (Sd)	12.8 (1.9)	*n* = 227
Platelet’s count (×100/mcL)—mean (Sd)	177.7 (96.9)	*n* = 226
Neutrophil count/mcL—mean (Sd)	4.3 (4.3)	*n* = 227
Lymphocyte count/mcL—mean (Sd)	1.6 (3.1)	*n* = 226
CRP mg/L—mean (Sd)	18.1 (23.2)	*n* = 217
AST IU/L—mean (Sd)	67.6 (64.8)	*n* = 226
ALT IU/L—mean (Sd)	42.4 (37.9)	*n* = 227
GGT IU/L—mean (Sd)	220.4 (207.4)	*n* = 227
ALP IU/L—mean (Sd)	172.8 (130.0)	*n* = 225
Total bilirubin mcmol/L—mean (Sd)	16.7 (12.8)	*n* = 225
Albumin g/L—mean (Sd)	36.4 (5.0)	*n* = 226
Creatinin mcmol/L—mean (Sd)	82.9 (43.4)	*n* = 227
Prothrombin time %—mean (Sd)	85.8 (17.0)	*n* = 224
AFP ng/mL—mean (Sd)	10,589 (68,631)	*n* = 224
ALBI—*n* (%)		*n* = 225
1/2/3	76 (33.8)/143 (63.6)/6 (2.7)	

Abbreviations: Sd, standard deviation; BMI, body mass index; HCC, hepatocellular carcinoma; MASH, Metabolic associated steatohepatitis; ECOG performance status, Eastern Cooperative Oncology Group Performance Status; BCLC, Barcelona Clinic Liver Cancer; TKIs, tyrosine kinase inhibitors; CRP, C-reactive protein; AST, aspartate aminotransferase; IU, international unit; ALT, Alanine aminotransferase; GGT, γ-glutamyl transpeptidase; ALP, alkaline phosphatase; AFP, alfa-fetoprotein; ALBI, albumin bilirubin.

**Table 2:** Performance of CRAFITY score for predicting treatment response. (**a**) Performance of the CRAFITY score for predicting response after the 3rd cycle, the 5th cycle, and beyond the 6th month of treatment. (**b**) Performance of the CRAFITY score after the 3rd cycle for predicting response after the 5th cycle and beyond the 6th month of treatment. (**c**) Performance of the CRAFITY score after the 5th cycle for predicting response beyond the 6th month of treatment.

(**a**)	
**Response to Treatment**	**CRAFITY Pre-Treatment**
**After the 3rd treatment cycle**	
CRAFITY—OR (95%CI)—*p*-value	
0	Ref
1	2.93 (1.06–8.13)—*p* = 0.0391
2	2.74 (0.91–8.22)—*p* = 0.0720
Overall *p*	0.1009
**After the 5th treatment cycle**	
CRAFITY—OR (95%CI)—*p*-value	
0	Ref
1	0.68 (0.30–1.56)—*p* = 0.3635
2	0.86 (0.32–2.27)—*p* = 0.7564
Overall *p*	0.6596
**Beyond the 6th month of treatment**	
CRAFITY—OR (95%CI)—*p*-value	
0	Ref
1	0.75 (0.29–1.96)—*p* = 0.5568
2	1.78 (0.55–5.77)—*p* = 0.3383
Overall *p*	0.3457
(**b**)	
**Response to Treatment**	**CRAFITY after the 3rd treatment cycle**
**After the 5th treatment cycle**	
CRAFITY—OR (95%CI)—*p*-value	
0	Ref
1	0.16 (0.06–0.46)—*p* = 0.0007
2	0.29 (0.09–0.99)—*p* = 0.0482
Overall *p*	0.0022
**Beyond the 6th month of treatment**	
CRAFITY—OR (95%CI)—*p*-value	
0	Ref
1	0.49 (0.18–1.36)—*p* = 0.1716
2	0.24 (0.04–1.30)—*p* = 0.0973
Overall *p*	0.1684
(**c**)	
**Response to Treatment**	**CRAFITY after the 5th treatment cycle**
**Beyond the 6th month of treatment**	
CRAFITY—OR (95%CI)—*p*-value	
0	Ref
1	0.53 (0.19–1.48)—*p* = 0.2283
2	0.13 (0.02–1.14)—*p* = 0.0656
Overall *p*	0.1219

Abbreviations: OR, odds ratio; CI, confidence interval; Ref, reference.

**Table 3:** Performance of the RECA score for predicting treatment response. (**a**) Performance of the RECA score for predicting response after the 3rd cycle, the 5th cycle, and beyond the 6th month of treatment. (**b**) Performance of the RECA score after the 3rd cycle for predicting response after the 5th cycle and beyond the 6th month of treatment. (**c**) Performance of the RECA score after the 5th cycle for predicting response beyond the 6th month of treatment.

(**a**)	
**Response to Treatment**	**RECA Pre-Treatment**
**After the 3rd treatment cycle**	
Mean (Sd)	
Non-responders	0.31 (0.34), *n* = 103
Responders	0.41 (0.31), *n* = 38
*p*-value	0.0970
AUC (CI95)	0.60 (0.50–0.70)
**After the 5th treatment cycle**	
Mean (Sd)	
Non-responders	0.28 (0.35), *n* = 99
Responders	0.33 (0.28), *n* = 41
*p*-value	0.4189
AUC (CI95)	0.53 (0.44–0.63)
**Beyond the 6th month of treatment**	
Mean (Sd)	
Non-responders	0.25 (0.32), *n* = 65
Responders	0.27 (0.32), *n* = 33
*p*-value	0.8124
AUC (CI95)	0.50 (0.38–0.62)
(**b**)	
**Response to Treatment**	**RECA after the 3rd treatment cycle**
**After the 5th treatment cycle**	
Mean (Sd)	
Non-responders	0.32 (0.36), *n* = 99
Responders	0.30 (0.34), *n* = 33
*p*-value	0.8421
AUC (CI95)	0.53 (0.42–0.64)
**Beyond the 6th month of treatment**	
Mean (Sd)	
Non-responders	0.26 (0.35), *n* = 62
Responders	0.18 (0.31), *n* = 25
*p*-value	0.3284
AUC (CI95)	0.57 (0.44–0.69)
(**c**)	
**Response to Treatment**	**RECA after the 5th treatment cycle**
**Beyond the 6th month of treatment**	
Mean (Sd)	
Non-responders	0.21 (0.32), *n* = 59
Responders	0.15 (0.31), *n* = 29
*p*-value	0.4143
AUC (CI95)	0.57 (0.44–0.69)

Abbreviations: Sd standard deviation; AUC, area under the curve; ratio; CI, confidence interval.

## Data Availability

The data related to this study are accessible upon approval from the administrators of Hôpital Saint-Joseph.

## Supplementary Materials

The following supporting information can be downloaded at: https://www.mdpi.com/article/10.3390/biomedicines14051043/s1, Supplementary Table S1. Patient characteristics prior to treatment with ICIs (Chinese multicenter external validation cohort); Supplementary Table S2. Therapeutic regimens in the Chinese validation cohorts; Supplementary Table S3. Multivariable Cox regression models for overall survival (Chinese Cohorts); Supplementary Table S4. Logistic regression for early response prediction (Chinese Cohorts); Table S5. Adverse events (AE) related to ICIs, with or without angiogenesis-targeting agents, in the French cohort (*n* = 229); Supplementary Figure S1. Flow diagram illustrating the outcomes for HCC patients included in the CRAFITY score assessment; Supplementary Figure S2. Prognostic performance of the RECA and CRAFITY scores for overall survival according to treatment regimens.

## Abbreviations

AFP	Alpha-fetoprotein
ALP	alkaline phosphatase
ALT	alanine aminotransferase
ALBI	Albumin-Bilirubin
AST	aspartate aminotransferase;
BCLC	Barcelona Clinic Liver Cancer
CIs	Confidence intervals
CP	Child–Pugh
CRP	C-reactive protein
CTLA-4	Cytotoxic T-lymphocyte antigen-4
DCR	Disease control rate
ECOG PS	Eastern Cooperative Oncology Group Performance Status
EV	Esophageal varices
GGT	γ-glutamyl transpeptidase
HRs	Hazard ratios
HCC	Hepatocellular carcinoma
ICIs	Immune checkpoint inhibitors
ITs	Immunotherapies
MASH	Metabolic associated steatohepatitis
RECIST	Response evaluation criteria in solid tumors
OR	Odds ratio
ORR	Overall response rate
OS	Overall survival
NCI-CTCAE	National Cancer Institute Common Terminology Criteria for Adverse Events
PR	Partial response
PD-1	Programmed death receptor-1
PD-L1	Programmed death ligand-1
(Q1, Q3)	Interquartile ranges
Ref	Reference
SD	Stable disease
SD	Standard deviations
TKIs	Tyrosine kinase inhibitors
VEGF	Vascular epidermal growth factor
