# Supplementary material for: Hepatocellular Carcinoma Treatment with Immune Checkpoint Inhibitors: RECA and CRAFITY Scores Reveal Distinct Clinical Courses and Highlight the Role of Systemic Inflammation in Prognosis
Source: Biomedicines. 2026 May 3;14(5):1043. doi: 10.3390/biomedicines14051043 (PMC13203940; doi:10.3390/biomedicines14051043)
Supplement: Supplementary file 1 [file biomedicines-14-01043-s001.zip › biomedicines-4204496-supplementary.pdf]

Supplementary Table S1. patient characteristics prior to treatment by ICIs (Chinese multicenter external validation cohort).

Baseline Characteristics	RECA Cohort (n = 332)	CRAFITY Cohort (n = 410)
Age, years—mean (Sd)	70.9 (9.6)	68.7 (8.9)
BMI—mean (Sd)	26.0 (5.3)	27.4 (5.0)
Male sex—n (%)	276 (83.1)	356 (86.8)
Cirrhosis—n (%)	281 (84.6)	325 (79.3)
Etiology of HCC—n (%)		
Alcohol/Virus B	43 (13.0) /240 (72.3)	38 (9.3) /315 (76.8)/
MASH/Other	48 (14.5)/1 (0.2)	44 (10.7)/13 (3.2)
Child–Pugh grade A—n (%)	265 (79.8)	352 (85.9)
Esophageal varices—n (%)	121 (36.4)	117 (28.5)
ECOG PS—n (%)		
0/1/2	132 (39.8) /163 (49.1)/37 (11.1)	199 (48.5)/184 (44.9)/27 (6.6)
Edmondson grade 3–4—n (%)	118 (35.5)	121 (29.5)
MTD, cm—mean (Sd)	6.4 (3.9)	5.6 (3.3)
Extrahepatic disease—n (%)	126 (38.0)	118 (28.8)
BCLC stage C—n (%)	241 (72.6)	249 (60.7)
Macrovascular invasion—n (%)		
VP 1/ 2; VP3	56 (16.9)/38 (11.4)	52 (12.7)/33 (8.0)
VP4; Other	31 (9.3)/17 (5.1)	22 (5.4)/19 (4.6)
No	190 (57.3)	284 (69.3)
Hemoglobin, g/dL—mean (Sd)	12.4 (1.9)	13.0 (1.8)
Platelets, $\times 10^9$/L—mean (Sd)	164.7 (101.3)	186.9 (91.6)
Neutrophils, $\times 10^9$/L—mean (Sd)	4.8 (4.6)	3.9 (3.8)
Lymphocytes, $\times 10^9$/L—mean (Sd)	1.4 (0.8)	1.7 (1.0)
CRP, mg/L—mean (Sd)	23.6 (26.4)	14.9 (18.7)
AST, IU/L—mean (Sd)	78.9 (71.2)	59.8 (54.1)
ALT, IU/L—mean (Sd)	45.6 (41.2)	39.2 (34.5)
Albumin, g/L—mean (Sd)	35.1 (5.3)	37.6 (4.6)
Total bilirubin, μmol/L—mean (Sd)	18.9 (14.1)	15.1 (11.3)
Creatinine, μmol/L—mean (Sd)	87.6 (46.8)	79.4 (39.7)

Abbreviations: Sd, standard deviation; BMI, body mass index; HCC, hepatocellular carcinoma; MASH, Metabolic associated steatohepatitis; ECOG PS, Eastern Cooperative Oncology Group Performance Status; MTD, Maximal tumor diameter; BCLC, Barcelona Clinic Liver Cancer; CRP, C-reactive protein; AST, aspartate aminotransferase; IU, international unit; ALT, Alanine aminotransferase.

Supplementary Table S2. Therapeutic regimens in the Chinese validation cohorts.

Variable	RECA Analysis set (n = 332)	CRAFITY Analysis set (n = 410)
Initial regimen: anti-VEGF + anti-PD-L1—n (%)	61 (18.4)	75 (18.3)
Initial regimen: anti-PD-1-based combinations (non-TKI)—n (%)	104 (31.3)	129 (31.5)
Initial regimen: anti-PD-1 + TKI—n (%)	167 (50.3)	206 (50.2)
Patients receiving any subsequent-line systemic therapy—n (%)	143 (43.1)	179 (43.7)

Supplementary Table S3. Multivariable Cox regression models for overall survival (Chinese Cohorts).

Analysis/Comparison	Adjusted HR	95% CI	*p* Value	Covariates Included
RECA high-risk vs VLR/LR/MR	2.31	1.74–3.06	<0.001	Age, sex, Child-Pugh class, BCLC stage, macrovascular invasion, extrahepatic disease, regimen class, subsequent-line therapy
CRAFITY score 1 vs 0	1.49	1.12–1.98	0.006	Age, sex, Child-Pugh class, BCLC stage, macrovascular invasion, extrahepatic disease, regimen class, subsequent-line therapy
CRAFITY score 2 vs 0	2.47	1.76–3.47	<0.001	Age, sex, Child-Pugh class, BCLC stage, macrovascular invasion, extrahepatic disease, regimen class, subsequent-line therapy
CRAFITY score trend per level	1.58	1.36–1.84	<0.001	Same covariate structure as above

Supplementary Table S4. Logistic regression for early response prediction (Chinese Cohorts).

Analysis/Comparison	Odds Ratio	95% CI	P Value
RECA high-risk vs VLR/LR/MR	0.87	0.58–1.31	0.50
CRAFITY score 1 vs 0	0.94	0.65–1.36	0.74
CRAFITY score 2 vs 0	0.79	0.51–1.23	0.30
CRAFITY trend per level	0.91	0.75–1.11	0.28

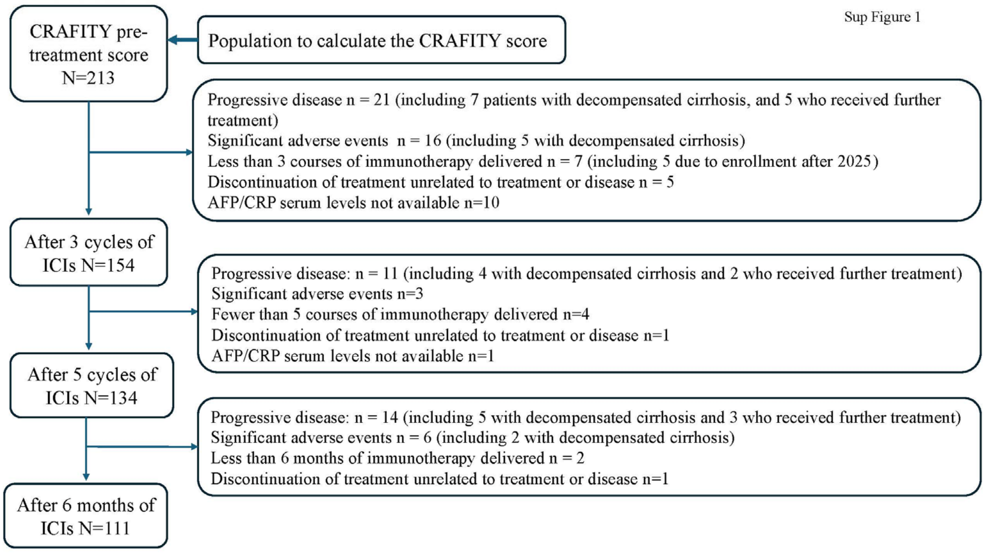

Supplementary Figure S1. Flow diagram illustrating the outcomes for HCC patients included in the CRAFITY score assessment (French Cohort).

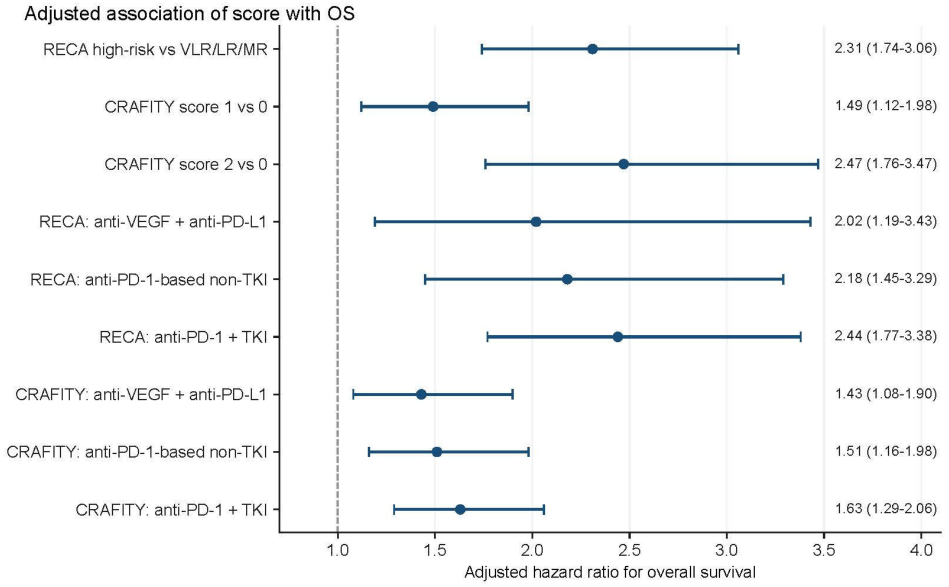

Supplementary Figure S2. Prognostic performance of the RECA and CRAFITY scores for overall survival according to treatment regimens (Chinese Cohorts).

Supplementary Table S5. Adverse events (AE) related to ICIs, with or without angiogenesis-targeting agents, in the French cohort (n=229)

**Treatment-related AE**	All grade AE	Grades ≥ 3
ICIs (N=229)		
**ICI-related AE n (%)**		
Patients with at least one event	159 (69.4)	39 (17.0)
Fatigue	110 (48.0)	8 (3.5)
Decreased appetite	32 (14.0)	8 (3.5)
Weight decrease	16 (7.0)	2 (< 1.0)
Pyrexia	9 (3.9)	1 (< 1.0)
Rash	21 (9.2)	3 (1.3)
Pruritus	30 (13.1)	2 (< 1.0)
Psoriasis	4 (1.8)	2 (< 1.0)
Toxic epidermal necrolysis	-	2 (< 1.0)
Colitis Diarrhea	42 (18.3)	5 (< 2.1)
Pancreatitis	2 (< 1.0)	-
Alanine aminotransferase increase/Hepatotoxicity	19 (8.3)	9 (3.9)
Hepatic function abnormal	16 (7.0)	13 (5.7)
Hypothyroidism	22 (9.6)	1 (< 1.0)
Hyperthyroidism	14 (6.1)	2 (< 1.0)
Adrenal deficiency	4 (1.8)	2 (< 1.0)
Arthritis	9 (3.9)	1 (< 1.0)
Polymyalgia	11 (4.8)	-
Polyneuropathy	1 (< 1.0)	1 (< 1.0)
Encephalitis	-	2 (< 1.0)
Pneumonitis	5 (2.2)	1 (< 1.0)
Myocarditis	2 (< 1.0)	1 (< 1.0)

Heart failure	12 (5.2)	3 (1.3)
AE leading to ICI interruption	77 (33.6)	
AE leading to ICI discontinuation	21 (9.2)	

Bevacizumab (N=155)		
**Bevacizumab-related AE n (%)**<br>Patients with at least one event	63 (40.6)	31 (20.0)
Esophageal varices hemorrhage	6 (3.9)	7 (4.5)
Upper gastrointestinal hemorrhage	-	6 (3.9)
Other Bleeding	9 (5.8)	7 (4.5)
Hypertension	17 (10.9)	2 (< 1.0)
Proteinuria	8 (5.2)	4 (2.6)
Thromboembolic event	5 (3.3)	2 (1.3)
Gastrointestinal complication	2 (1.3)	3 (1.9)
Wound healing complications	1 (< 1.0)	1 (< 1.0)
AE leading to bevacizumab interruption	56 (36.1)	
AE leading to bevacizumab discontinuation	21 (13.5)	

Eight patients (3.5%) died related to treatment for the following reasons: encephalitis (n=2), severe hepatitis (n=1), bleeding associated with clinical significant portal hypertension (n=2), bleeding associated with a peptic ulcer and ischemic heart disease (n=1), perforated gastric ulcer and liver failure (n=1), and esophagotracheal fistula (n=1; patient with a history of otorhinolaryngological radiation therapy).
